# Lung transplantation and organ allocation in Brazil: necessity or utility

**DOI:** 10.11606/S1518-8787.2019053000445

**Published:** 2019-02-20

**Authors:** Edison Moraes Rodrigues-Filho, Cristiano Augusto Franke, José Roque Junges

**Affiliations:** I Hospital de Clínicas de Porto Alegre. Centro de Tratamento Intensivo. Porto Alegre, RS, Brasil; II Universidade do Vale do Rio do Sinos. Programa de Pós-Graduação em Saúde Coletiva. São Leopoldo, RS, Brasil

**Keywords:** Lung Transplantation, legislation & jurisprudence, Bioethical Issues, Right to Health, Transplante de Pulmão, legislação & jurisprudência, Temas Bioéticos, Direito à Saúde

## Abstract

The philosophy of organ allocation is the result of two seemingly irreconcilable principles: utilitarianism and distributive justice. The process of organ donation and transplantation in Brazil reveals large inequalities between regions and units of the Federation, from the harvesting of organs to their implantation. In this context, lung transplantation is performed in only a few centers in the country and is still a treatment with limited long-term results. The allocation of the few organs harvested for the few procedures performed is defined mainly by chronology, a criterion that is not linked to necessity, which is a criterion of distributive justice, and neither to utility, a criterion of utilitarianism. This article reviews the organ allocation philosophy focusing on the case of lung transplantations in Brazil.

## RESUMO

A filosofia da alocação de órgãos é resultado de duas vertentes aparentemente inconciliáveis: utilitarismo e justiça distributiva. O processo de doação e transplante de órgãos no Brasil revela grandes desigualdades entre regiões e unidades da federação, desde a captação de órgãos até o implante dos órgãos. Nesse contexto, o transplante de pulmão é realizado em poucos centros no país e ainda é um tratamento, cujos resultados de longo prazo são limitados. A alocação dos poucos órgãos captados para os poucos procedimentos realizados é definida principalmente por meio da cronologia, um critério que nem é vinculado à necessidade, critério da justiça distributiva, e nem à utilidade, critério do utilitarismo. O presente artigo revisa a filosofia da alocação de órgãos com enfoque no caso dos transplantes de pulmão no Brasil.

## INTRODUCTION

The allocation of organs for transplantation follows two seemingly irreconcilable philosophical principles: distributive justice, based on the criterion of greatest need as priority, and utilitarianism, based on the criterion of greatest utility. Utility considers the life years gained with the procedure for a given patient. Some organ allocation models attempt to harmonize distributive justice and utilitarianism. Transplantations of solid organs and the organ allocation philosophy are a matter of fundamental importance in public health.

This article reviews the situation of lung transplantation and lung allocation in Brazil for adults, establishing a comparison with allocation in the United States of America (USA) and other countries, under the prism of the discussion between distributive justice and utilitarianism. The country's inequalities are highlighted, as questions and proposals regarding the role of distributive justice within a health system that seeks to prioritize universality and fairness are made.

### Organ Allocation Philosophy

The allocation of organs suffers significant influence of the utility criterion, which reflects the strength of utilitarianism in the last two centuries. Originated from the ideas of Jeremy Bentham and John Stuart Mill, utilitarianism applies an individual concept of happiness to the social level. This concept asserts that the greater or lesser happiness of life results from the difference between the sum of happy and unhappy moments. Utilitarianism transposes this concept to society, with the understanding that political decisions must achieve the highest degree of happiness for the greatest possible number of individuals, regardless of how it is distributed^1^. Though simple, this can allow a series of paradoxes, such as a portion of society being forced to live under levels of unhappiness that are intolerably unfair for a democratic state.

Allocation according to severity, on the other hand, is an application of distributive justice and of John Rawls’ concept of difference^2^. Distributive justice focuses on fairness in the distribution of goods and resources considered common, in an attempt to equalize the opportunities of access to these goods, using necessity as a criterion of justice to provide “to each that which they need”^3^.

Rawls developed a conception of justice in which all primary goods of a society (liberty and opportunity, income and wealth, and the foundations of self-respect) should be distributed equally unless an unequal distribution of any or all these goods is advantageous for the less favored^2^. Therefore, inequalities are allowed if they increase the initially equal shares of all, but are not allowed if, as in utilitarianism, they may reduce the smaller, initially balanced shares of some^2^ ^,^ ^4^.

The principles of the theory of justice developed by Rawls are as follows:

1. Each person should have equal right to the more extensive total system of basic liberties that are compatible with a system of similar freedoms for all;
2. Social and economic inequalities are to be arranged so that they are:
  - for the greater benefit of those with less benefits (principle of difference), and
  - linked to roles and positions that are open to all under conditions of fair equality of opportunity (principle of fair equality of opportunities).

Basic freedoms are the basic civil and political rights recognized in liberal democracies. The principle of difference is a priority in relation to the fair equality of opportunities, aiming to promote an ideal society, in which those who are arbitrarily “endowed” by social circumstances or natural talents reward the less favored^2^ ^,^ ^4^.

As at the bottom of Rawls’ reflection there is the premise that a well-ordered society would arise from an agreement of free, rational men, interested in their own welfare and responsible for the benefits of cooperation, the author proposed a theoretical exercise starting from an “original position” under a “veil of ignorance”^2^. The “veil of ignorance” has a procedural character to ensure the impartiality of the process and the fairness of the result. In this way, the individuals involved in the decision-making process would aim at minimizing disadvantages and not at maximizing advantages^5^.

Rawls’ philosophy depends on some hypothetical assumptions to achieve its three principles. The first is the original position, in which contractors are unaware of their social status, intellectual and physical skills. The second is that, in this condition, the rational choice would be for a society in which the worst alternative chosen would be the best possible, known as maximizing the minimum or maximin. The third is that societies would be stable and that, once a particular arrangement had been chosen, it would be irreversible^6^.

Rawls’ theory is a standardized theory of justice that grants resources according to each individual's needs. Other standardized theories would distribute goods according to other criteria, such as merit and social position. Non-standardized theories, on the other hand, rely solely on the legitimacy of the acquisition of certain goods^6^.

Organ and tissue transplants are a portrait of the techno-scientific advancement of the 20^th^ century. The first successful combined heart-lung transplantation was conducted in 1981, and the first successful lung transplantation was conducted in 1983^7^ ^,^ ^8^. The first double-lung transplantation was conducted in 1983^9^. Since then, the number of lung transplantations in the world grew exponentially^10^. However, lung transplantations are still limited by long-term results. This limitation is mainly due to the graft's chronic dysfunction, with special attention to the bronchiolitis obliterans syndrome, a phenomenon related to the manifestation of rejection in the airways^11^. Even with limitations, transplantation is indicated for a series of chronic lung diseases in final stage of evolution^12^.

### Lung Allocation for Transplantation in the World

The first American organ allocation attempt happened in 1984, when the Organ Procurement and Transplantation Network (OPTN) was created. This network monitors all patients listed for transplantation and is responsible for the harvesting, allocation and transplantation of solid organs in the whole country. There are 68 lung transplantation centers in the US and each is a member of one of the 62 Organ Procurement Organization (OPO) spread around the country^13^.

Between 1990 and 1995, lung allocation followed the chronological criterion from the inclusion of the receptor in the waiting list, according to their ABO blood type and anatomical compatibility (proportionality of the ribcage and height between donors and receptors)^14^. Since 1995, the peculiarity of patients with pulmonary fibrosis started being considered, as these patients may experience a rapid clinical deterioration, dying while waiting for transplantation. These patients started being granted a 90-day “bonus” when being included in the list^10^ ^,^ ^15^, that is, they are included in the list as if they had already been on it for 90 days. Currently, the lung transplantation list in the US is based on the Lung Allocation Score (LAS), established in 2005, a score that seeks to balance aspects related to the risk of death while on the waiting list and prognostic aspects related to post-transplantation survival rates. For this, a formula combines the death estimate of one year on the waiting list without transplantation (severity) with the number of days gained one year post-transplantation (utility)^16^.

Since the LAS's implementation, the number of transplantations has increased, although the number of donors has not. The diagnoses’ distribution changed, with more patients with pulmonary fibrosis being transplanted, in addition to an increase in the receptors’ age range^12^. There were also substantial declines in the waiting list and its death rates, without compromising the post-transplantation survival rates^17^ ^,^ ^18^. The LAS values have been progressively increasing, which is compatible with the greater severity of patients listed in the last 10 years^19^. Despite that, Crawford et al.^20^ recently evaluated data from the United Network for Organ Sharing (UNOS) pertaining to 3,548 transplantations conducted from May 2005 to March 2014, and observed an improvement in survival rates among receptors with higher scores. The OPTN implemented the first in-depth review of the LAS formula in February 2015. This review included modifications in the variables used in the LAS formula and in the relative weight of the variables used to estimate the risk of death in the next year without transplantation and in the first year post-transplantation^13^.

Germany introduced the LAS in 2014, which resulted in a reduction in mortality and in waiting time, especially for patients with cystic fibrosis and pulmonary hypertension^21^. Other countries used different forms of allocation. Switzerland introduced, in July 1, 2007, a type of allocation in which the priority is given to critical patients under mechanical ventilation or with extracorporeal support, followed by other severity criteria and, lastly, by patients with more time on the waiting list^22^. Prioritization according to necessity (severity) has also been adopted by France since 2007^23^. The Swiss and French models are more compatible with distributive justice.

Pure utilitarian models consider as better candidates patients who may gain more life years by procedure, subtracting the number of years offered by alternative treatments. In this way, the LAS score would me a mixed model that tries to harmonize distributive justice and utilitarianism. Valuing, at least partially, utility, reflects the tendency of many transplant surgeons towards the utilitarian philosophy, which may implicitly or explicitly influence their decisions, even in health systems guided by distributive justice. This happens because, even unconsciously, many transplant teams may choose less severe cases with greater chance of survival post-transplantation.

### Lung Transplantation in Adults and Organ Allocation in Brazil

In Brazil, the Brazilian Registry of Transplantations (RBT) of the Brazilian Association of Organ Transplantations (ABTO), estimated, in 2016, a need for 1,636 lung transplantations, of which only 92 were performed in this period, a small but growing number since 2011^24^. On December 2016, there were only 172 active adult patients on the waiting list. That same year, 127 new adult patients were admitted, but 43 deaths were listed. The high number of deaths indicates the severity of these patients and the low supply of organs. Of the 27 units of the federation, only the three states (Rio Grande do Sul, São Paulo and Ceará) with the country's six active teams registered inclusions in the list, which creates the need for patients to be linked to the existing teams, even if far from their state, to get a transplantation. The distribution of lung transplantations performed per million inhabitants (lmp) in 2016 by region was as follows: in the South, 1.2 lmp; in the Southeast, 0.6 lmp; in the Northeast, 0.1 lmp; in the Midwest, 0.0 lmp; and, in the North, 0.0 lmp. Therefore, approximately 90% of transplantations were performed in the South and Southeast regions, where a little more than 55% of the Brazilian population is distributed^24^. Logistical issues, especially related to the tolerance of the lungs outside the organism, prevent their harvesting at locations that are too distant from where they should be implanted^25^.

It is important to note that, although there is only one registry per person, the distribution of organs donated is conducted by the state, and later within the macro regions specified in Ordinance 2,600 of the Ministry of Health^26^. Only candidates with an active status on the waiting list may receive the offered organs. Many candidates are semi-active (incomplete registry, recent blood transfusions or no clinical conditions for transplantation) or inactive (registry has been outdated for more than three months) and do not compete, temporarily, for the organs offered^26^.

In addition to geographic disparities in the availability of transplantation teams, there are also disparities in organ harvesting, including in regions where lung transplantations are performed. ABB's RBT data from 2016 showed that the mean number of lmp with effective donors was 14.6. In the South region, with two active lung transplantation teams in Rio Grande do Sul, there were 30.1 lmp with effective donors in 2016, mainly due to the high performance of the state of Santa Catarina, which accounted for 36.8 lmp with effective donors. Paradoxically, in the Southeast region, with three active teams in the state of São Paulo, there were 15.5 lmp with effective donors, slightly above the national average. In the Northeast, with an active team in Ceará, there were 9.9 lmp with effective donors, below the national average^24^. It is important to point out that effective donors are those on whom the organ removal surgery is initiated. This does not necessarily include the removal of any organ, especially lungs, one of the offered organs with the lowest utilization rates, possibly lower than 5%^27^. In addition, some organs are discarded due to contraindications identified after their removal. Because time out of the body is crucial, the distance between the site of removal and the hospital where implantation will be performed is a significant factor. Thus, in the states where there are active teams, the proportion of transplantations was as follows: in Rio Grande do Sul, 3.1 lmp; in São Paulo, 1.1 lmp; and in Ceará, 0.7 lmp^24^. A complicating aspect of the reduced number of lung transplantations per year is that some teams perform below-ideal transplantations to achieve favorable outcomes^28^.

The health system in Brazil follows the necessity criterion, according to the health systems’ guidelines, which are based on the notion of universality and equity^29^. This model emerged in England with the creation of the National Health Service after World War II. With the Federal Constitution of 1988, Brazil started also following this premise, under the aegis of the Brazilian Unified Health System (SUS), which replaced the social security system based on the notion of merit (contributing worker). Currently, a supplementary health system based on market laws coexists with a public health system, SUS, which has a universalist inclination and is focused on fully meeting the population's health needs^29^.

In Brazil, lung transplantation candidates are listed according to the chronological criterion. However, depending on the teams’ severity assessment or due to clinical contraindications, patients may remain temporarily semi-active or inactive, or may be permanently removed from the list. Although the suitability of transplantation teams is not under discussion, this system, in addition to lacking transparence, may compromise the autonomy and justice of the process. When there is organ supply, active patients compete according to waiting list time, provided there is ABO blood group identification, ribcage size compatibility, updated immunological reactivity test and, eventually, ABO compatibility (in the absence of receptors with the same ABO blood group as the donor). Patients who receive an organ that does not function within the first 48 hours are an exception to the criteria above. In these situations, these receptors can be listed to receive another organ with urgency, being automatically placed at the top of the list^26^.

Finally, since we have very few active teams, all of which are geographically concentrated, there is a tendency for only organs offered near these centers to be used. In this way, more severe receptors close to other teams may lose the chance of receiving a compatible organ harvested in another region.

## DISCUSSION

The data available in Brazil show how inequalities related to the provision of health services may compromise the necessity criterion. It can be argued that, during the emergence of teams that are capable of performing such complex procedures, inequalities could be tolerated, as they would indirectly benefit individuals from unassisted regions, according to the principle of difference. However, few individuals have the necessary social and family structure to transplant organs in other regions or even in other states, since they need to be close to the medical teams to receive pre- and post-procedure care. It is also possible that the prerogative of teams to assess severity may be subjective and lead to unexpectedly unfair consequences over the candidates’ position on the waiting list. Subjective aspects could be circumvented with the application of the LAS. In any case, the allocation of lungs according to the chronological criterion is no longer an acceptable model, even considering the low number of transplantation teams in Brazil.

In a health system based on universality and equity, the application of the criterion of necessity, based on distributive justice, is ideal. However, it is difficult not to recognize that allocation based on a criterion that attempts to be an intermediate between need and utility may be attractive for a procedure involving great public investment. Perhaps the dichotomy between necessity and utility is artificial, for there is no real distributive justice when there are no organs for all who need them. In addition, the application of a system that, at least in part, privileges need, cannot be considered totally utilitarian.

While it is hard to imagine that distributive justice and utilitarianism can be harmonized, it is possible that a score like the LAS approaches that purpose. Perhaps, a score that considers the post-transplant prognosis only is maximizing, rather than utilitarian.

On the other hand, a score that integrates life years gained post-transplantation and waiting list mortality for a given patient, and that does not take into account the outcome of other patients, may have a utilitarian bias. Is it possible to use partial or totally utilitarian scores without violating the principles of universality and equity, embodied in the Federal Constitution of 1988? Even if it hurts the principles of our health system, isn't abandoning the chronological criterion long overdue? The chronological criterion is clearly inadequate as it compromises the transparency of the process.

In fact, our greatest limitation is still not practicing equity, because our health system does not meet the basic requirement of treating the unequal unequally to promote equity. The unequal distribution of access to transplantation workers treats the unequal equally, or worse, treats the unequal unequally, but does not promote equity. One possibility is that our system will practice the principle of difference even more by creating special incentives for the emergence of new transplantation teams in states where they are needed. Another alternative is to invest in the approximation of donors to existing teams, considering the need for a greater volume of cases to achieve better results.

Even if it is possible to harmonize need and utility, we need to further increase distributive justice in Brazil. A health system based on universality and equity is something that needs to be built. Recalling Rawls's words, quoted by Michael Sandel^30^: “we must repudiate the claim that if institutions are failures because circumstances are unjust, that injustice must inevitably be transferred to human providences. Eventually this reflection is used as an excuse to ignore injustice, as if refusing to accept injustice is the same as being unable to accept death” (p. 177).

## FINAL CONSIDERATIONS

Perhaps the decision to accredit new lung transplantation teams can no longer be made under a veil of ignorance in relation to the country's inter- and intra-regional inequalities. Justice cannot be blind to such matters, as in the depiction of the Roman goddess *Justitia* with a blindfold over her eyes. When establishing and applying the rules of the game, it is reasonable to choose a system that prioritizes the patients who most need it. The establishment of these rules must encompass healthcare as a whole, for health belongs to the basic structure of society, which is the central focus of Rawls’ theory of justice. In addition, it is important to evaluate the performance of the teams according to other criteria besides survival results after one and five years of the transplantation, since this evaluation is utilitarian only. For example, number of procedures, waiting list time and mortality should also be considered.

The persistence of the centralization of the new accreditations of lung transplantation teams may perpetuate inequalities from the point of view of distributive justice, that is, of distribution according to necessity. Thus, other standards of justice may be inadvertently stimulated. It may be so that “distribution according to social position,” an aristocratic formula of justice, is being used as a standard. This may be occurring because, as there is concomitance of the public and supplementary system, patients treated by the latter are likely to have more socioeconomic conditions to stay close to the transplantation teams, both pre- and post-transplantation. In addition, the concomitance of remuneration by the public and by the supplementary or private health system is also a stimulus to a view of justice that is not based on standards, but on the legality of the process only. This last aspect sometimes oscillates the pendulum of justice towards the use of standards, not necessarily necessity, and at other times, towards the absence of standards.
